# Silicon mitigates iron deficiency in two energy cane cultivars by modulating physiological and nutritional mechanisms

**DOI:** 10.3389/fpls.2023.1204836

**Published:** 2023-05-31

**Authors:** Gelza Carliane Marques Teixeira, Renato de Mello Prado, Antonio Márcio Souza Rocha, Murilo Bassan Princi, Caio Soares de Andrade

**Affiliations:** ^1^Laboratory of Plant Nutrition, Department of Agricultural Sciences, São Paulo State University (UNESP), Jaboticabal, São Paulo, Brazil; ^2^Laboratory of Biogeochemistry, Department of Technology, São Paulo State University (UNESP), Jaboticabal, São Paulo, Brazil

**Keywords:** abiotic stress, beneficial element, defense mechanisms, modulating agent, nutritional efficiency

## Abstract

**Introduction:**

Alkaline soils with iron (Fe) deficiency are found in many regions of the world, and the use of silicon (Si) can mitigate the damages caused by such deficiency. The aim of this study was to evaluate the effect of Si in mitigating a moderate deficiency of Fe in two energy cane cultivars.

**Methods:**

Two experiments were performed, one with the VX2 cultivar and the other with the VX3 cultivar of energy cane, which were cultivated in pots with sand and a nutrient solution. In both experiments, treatments followed a factorial scheme 2x2, designed based on the sufficiency and deficiency of Fe, being combined with the absence or presence of Si (2.5 mmol L^-1^), disposed in a randomized blocks design with six replicates. In the condition of Fe sufficiency, plants were cultivated in a solution containing 368 µmol L^-1^ of Fe, while plants cultivated under deficiency were initially submitted to cultivation with a 54 µmol L^-1^ concentration of Fe for 30 days, and later, with Fe complete omission for 60 days. The supply of Si was carried out by applying 15 fertirrigations with Si (via root and leaf) during the initial stage of seedling development, and after transplanting, the nutrient solution was added daily (via root).

**Results and discussion:**

Both cultivars of energy cane were sensitive to Fe deficiency in the absence of Si, impairing its growth by causing stress and pigment degradation, thus reducing the photosynthesis efficiency. The supply of Si mitigated the damages caused by Fe deficiency in both cultivars, by increasing Fe accumulation in new and intermediate leaves, stem, and roots in the VX2 cultivar, and in new, intermediate, and old leaves and stem in the VX3 cultivar, which in turn reduced stress and favored both the nutritional and photosynthesis efficiency, while increasing the dry matter production. Si by modulating physiological and nutritional mechanisms, mitigates Fe deficiency in two energy cane cultivars. It was concluded that Si can be used as a strategy to improve growth and nutrition of energy cane in environments that are susceptible to Fe deficiency.

## 1 Introduction

Global sugarcane production has increased over the last decades due to its importance as a renewable energy source regarding the production of ethanol fuel (Hoang et al., 2015). Brazilian bioethanol is one of the most prominent biofuels produced on a large scale, representing 30% of the global fuel ethanol production share. Around 8.6 billion gallons were produced in 2019, second only to the US fuel ethanol production in the same period (15.8 billion gallons) (Karp et al., 2021).

The cultivation of energy cane (*Saccharum spontaneum* L.) has expanded a production the electric energy because which has a higher energy conversion in comparison to sugarcane, due to its higher dry matter production and fiber (Matsuoka, 2017). Energy cane is classified according to its composition and purpose of use of the raw material (aptitude), namely type 1: above 15% sucrose and 18% fiber, destined mainly for the production of 2^nd^ generation ethanol; and type 2: sucrose less than 6% and fiber above 28%, destined for energy generation (Matsuoka et al., 2014).

Another characteristic that contributes for the increasing areas of energy cane cultivation is its higher tolerance to adverse environments, being frequently cultivated in soils with low fertility (Carvalho-Netto et al., 2014). However, one of the limitations for increasing the global agriculture production regards the abundance of alkaline soils, which constitute 30% of the world’s agricultural soils (Guerinot and Yi, 1994), given that these soils present low availability of micronutrients, such as iron (Fe) (Abbaszadeh-Dahaji et al., 2020). The uptake and availability of Fe to plants is frequently insufficient, particularly in calcareous soils with low Fe availability. This is due to the high pH value, which results in the non-absorption of poorly soluble forms of Fe [Fe(OH)_3_] by the plants, causing nutritional disturbance and losses in crop yield and nutritional quality (Zuo and Zhang, 2011).

Hence, crops cultivated in this type of soil induce moderate or severe deficiencies depending on the species or cultivar that is used (Gonzalo et al., 2013). The response of energy cane (type 1 and 2) in this environment remains unknown. It can be estimated that tolerance varies between cultivars, where the cultivar with the highest fiber production (type 2) could be more rustic and have more efficient Si uptake mechanism, which could result in greater tolerance to Fe deficiency.

Soil Fe deficiency causes several biological damages to plants, such as increased oxidative stress (Nikolic et al., 2019), which induces characteristic symptoms such as interveinal yellowing in the surface of young leaves, known as chlorosis (de Mello Prado, 2021). Therefore, supplying Fe could avoid chlorophyll degradation and even induce its synthesis, as this microelement induce the formation of its precursor porphyrin (Becker et al., 2020) and consequently increases the chlorophyll content and the quantum efficiency of photosystem II (PSII) (Bertamini et al., 2002; Yadavalli et al., 2012) and plant’s growth.

A strategy that is commonly adopted to reduce damage caused by nutritional deficiencies is the use of silicon (Si), an element that is benefic to plants by acting in several mechanisms, such as the preservation of photosynthetic pigments observed in sorghum plants (Teixeira et al., 2020a) and barley plants (Nikolic et al., 2019) under Fe deficiency. The use of Si in sorghum cultivated under Mn deficiency promoted a reduction of oxidative stress and an increase in the use efficiency of this micronutrient, favoring dry matter production (De Oliveira et al., 2019; Oliveira et al., 2020).

The mechanisms involved in the mitigation of damages caused by Fe deficiency from the application of Si in energy cane plants, which acts as an accumulating organism (Ma and Takahashi, 2002), have not yet been demonstrated. We hypothesized that (i) it occurs by increasing Fe accumulation, mainly in new and intermediate leaves, and the nutritional efficiency, which might mitigate stress due to the reduction of the cells’ electrolytes leakage, when increasing the antioxidant compound carotenoid and consequently, the chlorophyll content, photosynthesis efficiency and dry matter production, and (ii) that these effects vary with the energy cane cultivar used due to its different attributes (sucrose or fiber production). If the hypothesis is corroborated, the use of Si in energy cane crops will be made possible in soils with Fe deficiency, which will have a relevant impact worldwide due to the global tendency in increasing renewable energy sources from energy cane biomass.

Thus, the aim of this study was to evaluate the capacity of Si application in mitigating stress caused by moderate Fe deficiency in two cultivars of energy cane, one with double aptitude to produce sucrose and fiber (VX3 - type 1) and the other only for the production of fiber (VX2 - type 2).

## 2 Material and methods

### 2.1 Growth conditions and plant material

**Figure 1:**
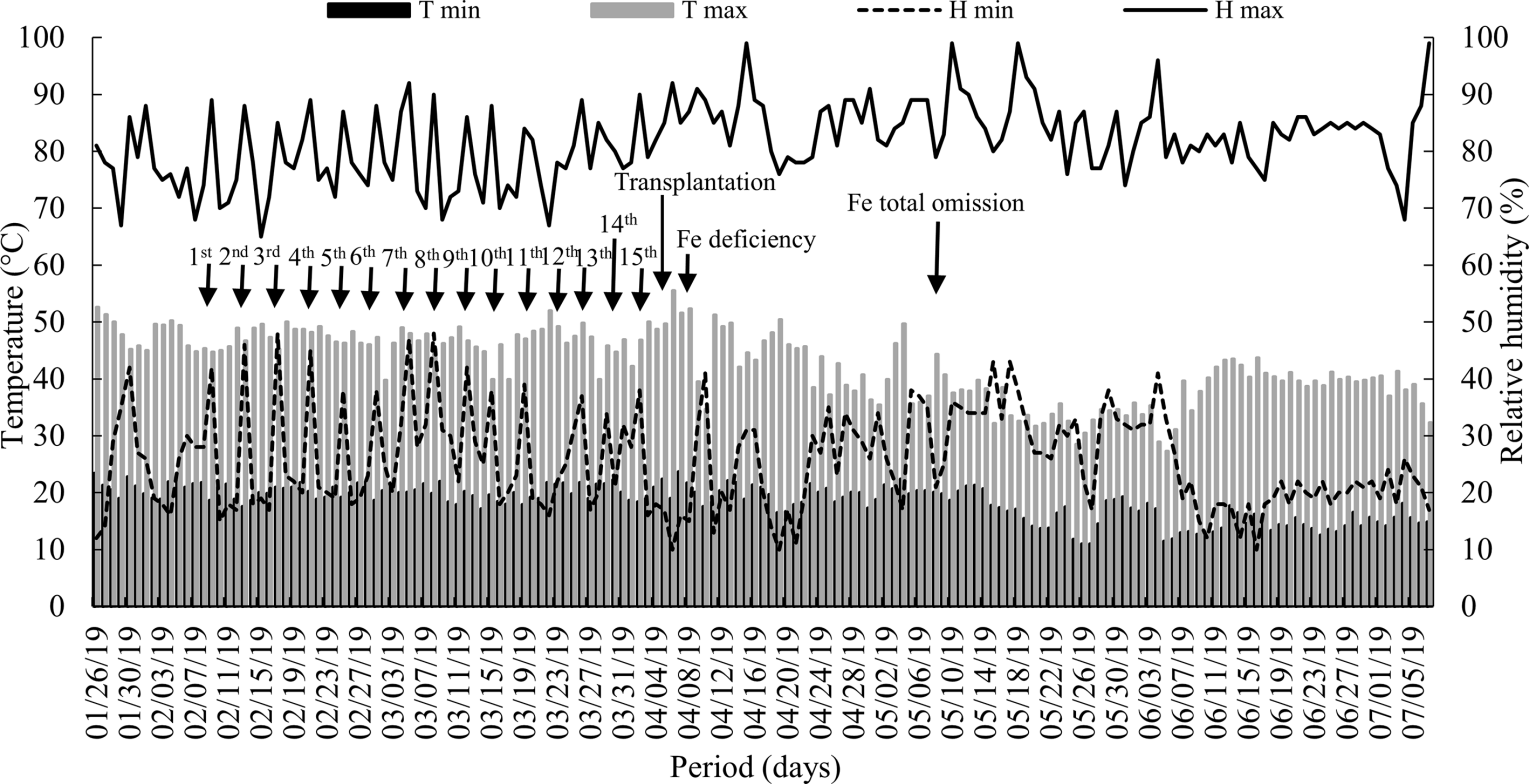
Maximum and minimum (T max and T min) air temperature and relative humidity (H max and H min) inside the greenhouse throughout the experimental period. Arrows indicate the dates: silicon supply, transplantation, start of iron deficiency, and iron total omission in nutrient solution. Fe = iron.

Two experiments were conducted simultaneously in a greenhouse at the São Paulo State University (UNESP), Campus of Jaboticabal/SP, from January to July 2019. The temperature and relative humidity inside the greenhouse were monitored and recorded daily with the aid of a thermo-hygrometer (**Figure 1**).

We used two energy cane cultivars the VX2 cultivar (type 2) of high fiber content (>28%) (Experiment 1), and the VX3 cultivar (type 1) of medium fiber content (>18%) (Experiment 2) (Matsuoka et al., 2014).

Both cultivars were obtained from the Brazilian Agricultural Research Corporation of the Ministry of Agriculture, Livestock, and Food Supply. All plant studies were carried out following relevant institutional, national, or international guidelines and regulations. Our research was not conducted with endangered species and was conducted following the Declaration of IUCN Policy on Research Involving Endangered Species.

### 2.2 Treatments and experimental design

The experiment was carried out in a 2 × 2 factorial scheme. The treatments consisted of sufficiency and deficiency of Fe; combined with either the absence (-Si) or the presence of silicon (+Si). Treatments were arranged in experimental design was randomized blocks, with six replicates.

Initially, mini stalks were planted in seedlings production trays filled with fine vermiculite. The nutrient solution used in this study was carried out as indicated by Hoagland and Arnon (1950), with ionic strength of 25%, over 7 days. Then, the nutrient solution was applied following the treatments with ionic strength adjusted to 50% until the end of the experiment. The ionic strength variation was performed to avoid salinity stress to the plants. The nutrient solution at full concentration was: 7.5 mmol L^-1^ of N, 0.5 mmol L^-1^ of P, 3.0 mmol L^-1^ of K, 2.5 mmol L^-1^ of Ca, 1.0 mmol L^-1^ of Mg, 1.0 mmol L^-1^ of S, 4.5 µmol L^-1^ of Mn, 23.0 µmol L^-1^ of B, 0.36 µmol L^-1^ of Zn, 0.15 µmol L^-1^ of Cu and 0.05 µmol L^-1^ of Mo. Sources used to supply nutrients: N: KNO_3_ and Ca(NO_3_)_2_.4H_2_O; P: KH_2_PO_4_; K = KH_2_PO_4_, KNO_3_ and KCl; Ca: Ca(NO_3_)_2_.4H_2_O; Mg and S: MgSO_4_.7H_2_O; Mn: MnCl_2_.4H_2_O; B: H_3_BO_3_; Zn: ZnCl_2_; Cu: CuCl_2_; and Mo: H_2_MoO_4_.H_2_O.

During this initial phase of seedling production, the concentration of Fe supplied in the nutrient solution was 368 µmol L^-1^ using a chelating Fe source (Fe-EDDHA = ethylenediamine-N,N′-bis(2-hydroxyphenylacetic acid) at 6% (83.33 g L^-1^) (Cavalcante et al., 2016).

The nutrient solutions were prepared daily with distilled and deionized water. The volume applied was defined from a preliminary test of substrate saturation to avoid losses by leaching, in which the adequate volume to be applied was found to be 10 mL per seedling, with the aid of a beaker. The pH value was maintained at 5.5 ± 0.2 and monitored daily with a digital pH meter (ICEL PH-1500). When necessary, it was adjusted with HCl or NaOH solution (1.0 mol L^-1^).

During this phase the seedlings were also enriched with Si, being applied the concentration of Si used was 2.5 mmol L^-1^ avoiding polymerization, seen that it starts at a concentration of 3.0 mmol L^-1^ of Si (Birchall, 1995). The source of soluble Si used in this study was sodium and potassium silicate stabilized with sorbitol (113.4 g L^-1^ of Si and 18.9 g L^-1^ K_2_O, 60,5 g L^-1^ de Na_2_O e 20 mL L^-1^ de sorbitol, at pH 11.8). Fifteen Si fertirrigation events were performed considering 4-days intervals, starting 10 days after full sprout emergence (DAE) (**Figure 1**), by applying 10 mL per seedling via substrate and 1.5 mL per seedling via foliar spraying, using a hand sprayer. The pH value of the solution containing fertirrigated Si was adjusted to 5.5 ± 0.2, in order to increase monomeric Si species in the solution, which is the absorbable form of the element by plants (Kudryavtsev and Figovsky, 2016). The amount of potassium present in the Si source was balanced in treatments that did not have the element, using a KCl solution (1.0 mol L^-1^), applied in the roots and leaves in the seedling production phase and via roots after transplanting.

At 70 DAE, seedlings were transplanted into 7.0 dm^3^ pots filled with 5.5 dm^3^ of sand that was previously washed with potable water, then with an HCl solution (1%) and finally deionized water. At transplantation, the seedlings had six completely developed leaves and the cut was made 30 cm from the last completely developed leaf sheath, i.e. close to one third of the leaves were removed. This procedure was performed because it is a common practice in seedling nurseries of energy cane, which aims at reducing water losses by transpiration at the time of transplantation to the soil (Landell et al., 2012).

Four days after transplantation, plants were submitted to the condition of nutritional deficiency, by means of modifying the Fe concentration in the solution of Hoagland and Arnon (1950), while for the sufficiency condition, the Fe concentration was maintained at 368 µmol L^-1^ (Cavalcante et al., 2016). The deficiency condition was initiated with a Fe concentration of 54 µmol L^-1^, corresponding to 60% of the recommended by Hoagland and Arnon (1950). After 30 days, in plants under the deficiency condition, the Fe was not included in the nutrient solution for a period of 60 days until plants were sampled.

After transplanting, plants cultivated in the presence of Si (+Si) continued to receive the element in the nutrient solution (2.5 mmol L^-1^). The Si supply was continuous throughout the phase of imposing the nutritional deficiency of Fe. The pH value of the nutrient solution was daily adjusted (5.5 ± 0.2), before being applied to the pots, and this solution was supplied constantly throughout the experimental period. The nutrient solutions also were prepared daily with distilled and deionized water. On a weekly basis, the pots received deionized water to wash the substrate, thus avoiding the accumulation of salts.

### 2.3 Variables analyzed

#### 2.3.1 Quantum efficiency of the photosystem II (Fv/Fm)

The quantum efficiency of the photosystem II-PSII (Fv/Fm) was estimated by measuring the chlorophyll’s fluorescence, performed between 7h and 8h. At this time, leaves were submitted to an adaptation period to the darkness, using tweezers of the device for a period of 30 min. The Fv/Fm was assessed by means of an excitation triggered by a pulsing red light (intensity of saturation of 3500 µmol). Measurements were performed on the first completely developed leaf, that is in the most recently developed leaf of each plant, and determined with the aid of a portable fluorimeter (Opti-Sciences - Os30P) (Lichtenthaler et al., 2005).

#### 2.3.2 Chlorophyll and carotenoids contents

Five 6-mg leaf discs were collected in the middle third of the leaf blade of the fourth completely developed leaf. The samples were submerged in 80% acetone in flasks previously covered with aluminum foil, remaining in this solution until complete discoloration. The readings were performed in a spectrophotometer (Beckman DU 640) at 663 nm for chlorophyll a (Cha), 647 nm for chlorophyll b (Chb) and 470 nm for carotenoids (Lichtenthaler, 1987). The values obtained were used to calculate pigment concentration, expressed in mg g^-1^ of fresh mass (FM).

#### 2.3.3 Electrolyte leakage index

Ten leaf discs (26.4 mm^2^ each) were collected from the first completely developed leaf and emerged into deionized water for 2 hours, and the electrical conductivity (EC_1_) of the solution was measured using a conductivity meter (TDS-3). Samples were autoclaved at 121°C for 20 min, and after cooling, the final electrical conductivity (EC_2_) was obtained. The electrolyte leakage index of was determined considering the equation: EC_1_/EC_2_ x 100 (Dionisio-Sese and Tobita, 1998).

#### 2.3.4 Dry matter production

The plants were separated into old leaves, intermediate leaves, new leaves, stems and roots, and then these were washed sequentially with water, detergent (Extran ^®^ - alkali) solution (0.1% v/v) (1 mL L^-1^), HCl solution (0.3% v/v) (3 mL L^-1^) and deionized water. The plant material was dried in an oven with air circulation (65.0 ± 5.0°C) until constant mass, and the dry matter of each plant was obtained by weighting the samples.

#### 2.3.5 Concentration of Si and Fe

The Si concentration was analyzed in old, intermediate, and new leaves, stems and roots. For such, 0.1 g of dry and ground material was added to 50 mL polyethene tubes. The samples were moistened with 2 mL of hydrogen peroxide (H_2_O_2_), the tube was placed in an oven at 95°C. After 30 min, the tubes were removed, and 4 mL of 50% NaOH were added to warm samples. The sample tubes were then gently vortexed and returned to the oven (95°C) for 4 h (Kraska and Breitenbeck, 2010). The Si concentration was determined by colorimetry using 1 mL extract plus 19 mL of water, 1 mL of HCl (1:1), and 2 mL ammonium molybdate. After 5 min, 2 mL of oxalic acid were added. The reading was performed by a spectrophotometer at 410 nm (Korndörfer et al., 2004).

The concentration of Fe in each organ of the plant was determined by the digestion of samples of plant material, using a digestive mixture of perchloric and nitric acid (1:2), with readings of Fe performed in spectrophotometry atomic absorption with air-acetylene flame (Bataglia et al., 1983).

Based on the Si or Fe concentration in the dry matter of plants, the accumulation of Si and Fe was calculated for each organ of the plant, according to the equation:


$$\text{Si or Fe Accumulation }\left(\text{mg per plant}\right)=\text{dry matter }\left(\text{g}\right)\text{ x Si or Fe concentration }\left({\text{g kg}}^{-1}\right)$$

#### 2.3.6 Efficiency in the uptake, translocation, and utilization of Fe

The nutritional efficiencies of Fe in the plants were obtained using accumulation data of this micronutrient and the plants’ dry matter. For this purpose, the following equations were used (Fageria and Baligar, 2005):


$$\text{Uptake efficiency }\left({\text{mg g}}^{-1}\right)=\text{Fe accumulation in the whole plant }\left(\text{mg per plant}\right)÷\text{ dry matter of root}\left(\text{g}\right)$$

Translocation efficiency (%)=(Fe accumulation in the aerial part (mg per plant)÷ Fe accumulation in the whole plant (mg per plant)) x 100

$$\text{Utilization efficiency }\left({\text{g mg}}^{-1}\right)={\text{ dry matter of whole plant}}^{2}\;÷\text{ Fe accumulation in the whole plant }\left(\text{mg per plant}\right)$$

### 2.4 Statistical analysis

Data were analyzed independently for each experiment, that is, there was no intention to statistically compare the effects on cultivars of energy cane. The data were submitted to a bidirectional variance analysis by the F test (p<0.05), after meeting the premises of normality (Test W of Shapiro-Wilk) and variance homogeneity (Test of Bartlett). A factorial analysis was used to test the principal effects of treatments with Si and Fe, as well as its interactions. Means were compared by the Tukey’s test at a 5% probability level, using the software SAS^®^ (Cary, NC, USA).

The data were also subjected to a hierarchical cluster analysis. For this purpose, data were standardized by the following equation: Zij = (Xij-Xj)/Sj, wherein j = number of variables; i = number of treatments; Zij = standardized value of Xij; Xj and Sj = mean and standard deviation of the variables, respectively. The Euclidean distance was used as a similarity coefficient and the UPGMA method (unweighted pair-group method using arithmetic averages) as a group connection algorithm (Teixeira et al., 2020b). The statistical tests were performed using the free software environment R and the package “pheatmap”.

## 3 Results

**Figure 2:**
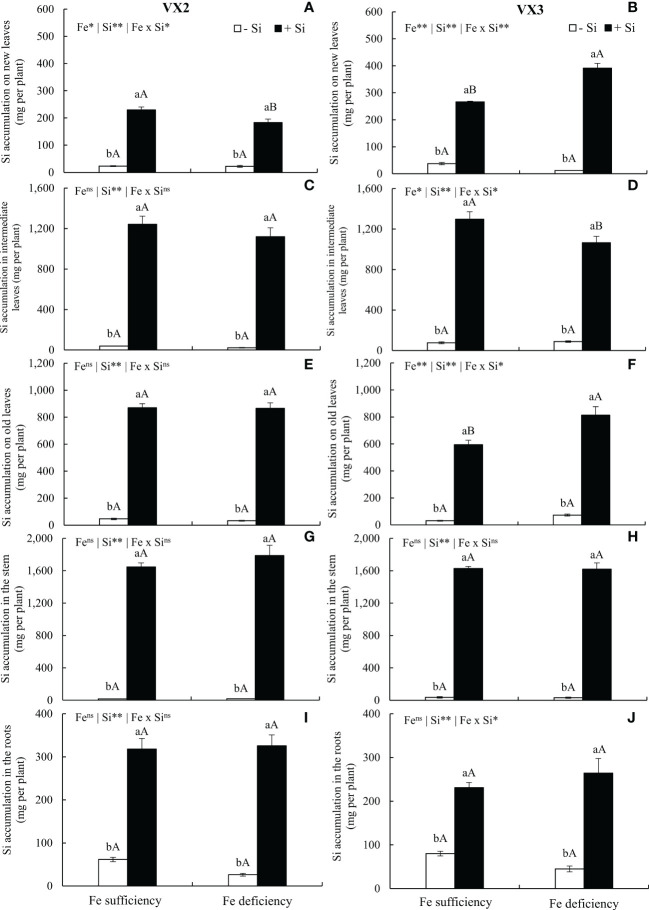
Accumulation of silicon (Si) in new **(A, B)**, intermediate **(C, D)**, and old leaves **(E, F)**, in stems **(G, H)** and roots **(I, J)** in both sufficiency and deficiency of iron (Fe) conditions, associated with the presence (+Si) and absence of Si (-Si) in plants of two cultivars of energy cane (VX2 and VX3). **, *significant at 1% and 5% probability levels, respectively; ^ns^not significant by the F-test. Lowercase letters indicate significant differences in relation to Si in the same Fe condition, while uppercase letters relates to Fe in the same condition of Si by the Tukey’s test. Bars represent the standard error of means (n = 6).

The supply of Si via nutrient solution increased its concentrations in all parts of the plants, cultivated under both sufficiency and deficiency of Fe, in both cultivars of energy cane. The amount of accumulated Si varied between cultivars, and the following order of accumulation for VX2 was stems > intermediate leaves > old leaves > roots > new leaves, while for VX3 it was stems > intermediate leaves > old leaves > new leaves > roots (**Figures 2A–J**).

**Figure 3:**
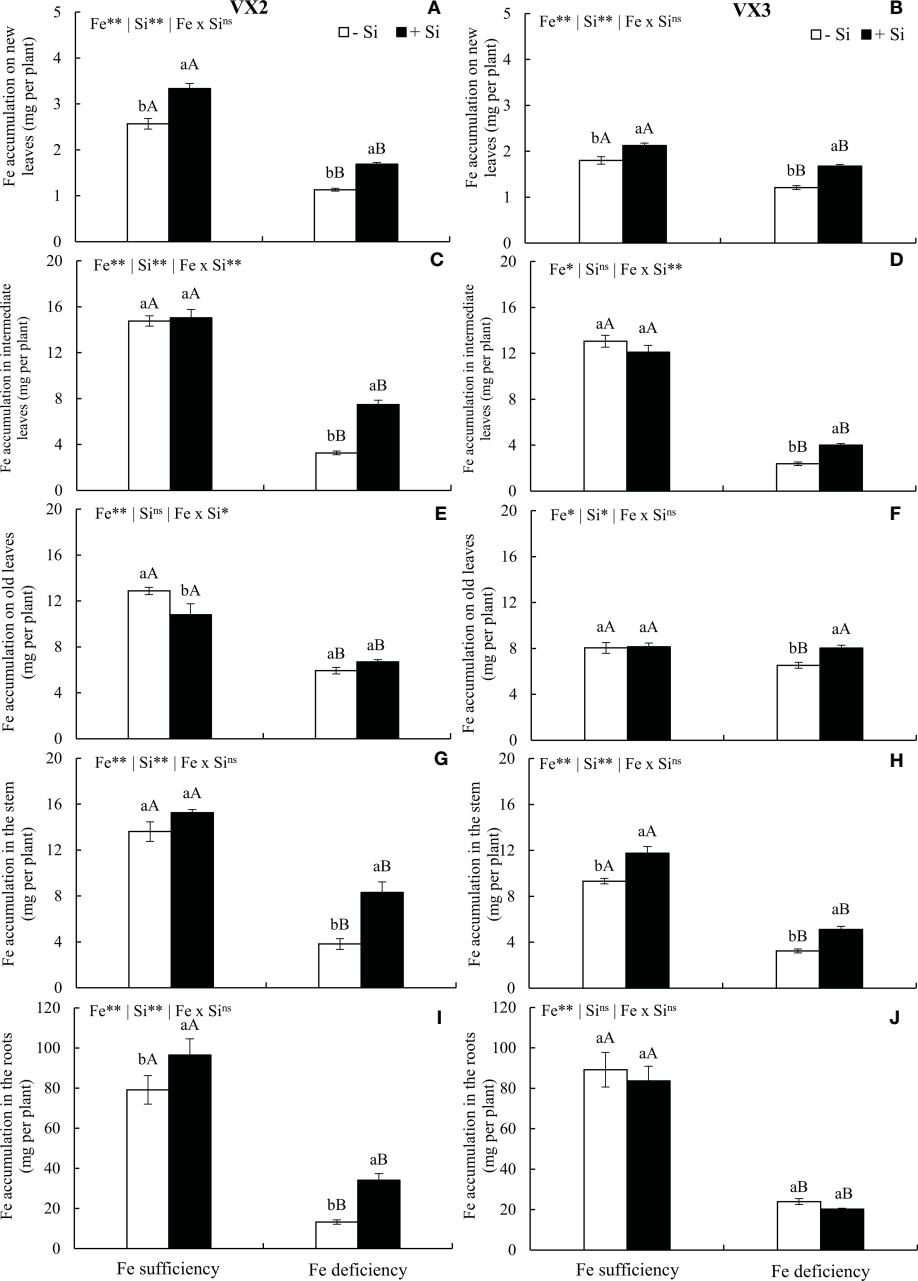
Accumulation of iron (Fe) in new **(A, B)**, intermediate **(C, D)** and old leaves **(E, F)**, in stems **(G, H)** and roots **(I, J)** in both sufficiency and deficiency of Fe conditions, associated with the presence (+Si) and absence (-Si) of silicon (Si) in plants of two cultivars of energy cane (VX2 and VX3). **, *significant at 1% and 5% probability levels, respectively; ^ns^not significant by the F-test. Lowercase letters indicate significant differences in relation to Si in the same Fe condition, while uppercase letters relates to Fe in the same condition of Si the Tukey’s test. Bars represent the standard error of means (n = 6).

In plants cultivated under the absence of Si, the moderate deficiency of Fe reduced the accumulation of the micronutrient in all parts of the plants of both cultivars, in comparison to the condition of Fe sufficiency and -Si. However, the addition of Si provided an increased accumulation of Fe in new and intermediate leaves, stem, and roots of the VX2 cultivar, and new, intermediate, and old leaves and stems of the VX3 cultivar, under ferric deficiency. However, such an elevated Fe accumulation in the entire plant was 110% greater in VX2 in comparison to VX3, in the Fe deficiency condition (**Figures 3A–J**). The Fe accumulation in each part of the plants was changed with Si supply and nutritional deficiency in a specific manner for each cultivar. The roots presented higher Fe accumulation, while new leaves displayed the lowest values (**Figures 3A–J**).

**Figure 4:**
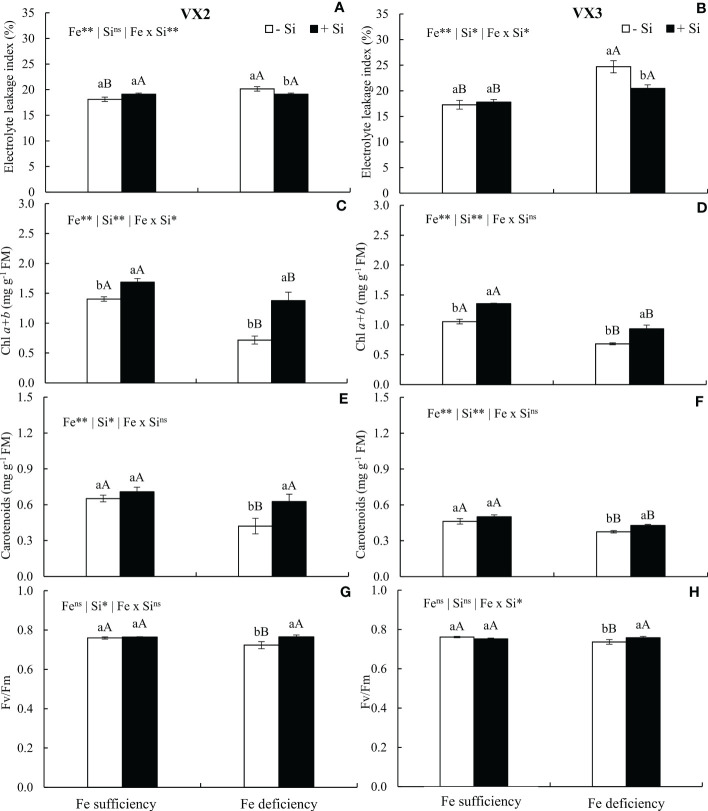
Electrolyte leakage index **(A, B)**, total chlorophyll content (Chl *a+b*) **(C, D)**, carotenoids **(E, F)**, and quantum efficiency of FSII (Fv/Fm) **(G, H)** in both sufficiency and deficiency of iron (Fe) conditions, associated with the presence (+Si) and absence (-Si) of silicon (Si) in plants of two cultivars of energy cane (VX2 and VX3). **, *significant at 1% and 5% probability levels, respectively; ^ns^not significant by the F-test. Lowercase letters indicate significant differences in relation to Si in the same Fe condition, while uppercase letters relates to Fe in the same condition of Si the Tukey’s test. Bars represent the standard error of means (n = 6).

The reduction in the Fe content in the foliar tissue induced a stress condition, observed by the increased index of cellular electrolytes leakage index, especially in plants cultivated in the absence of Si. Nevertheless, the supply of the Si reduced the cellular electrolyte leakage index in the condition of Fe deficiency in both cultivars, in a similar manner to the results observed in plants cultivated under Fe sufficiency, considering VX2 plants (**Figures 4A, B**).

The deficiency in Fe reduced the concentrations of chlorophylls and carotenoids, but the addition of Si in the nutrient solution resulted in increased amounts of these photosynthetic pigments in both cultivars (**Figures 4C–F**). The increment observed for these pigments reflected in an increased photosynthetic efficiency, which was expressed by the values of Fv/Fm, and remained similar to the values observed in the condition of Fe sufficiency (**Figures 4G, H**).

**Figure 5:**
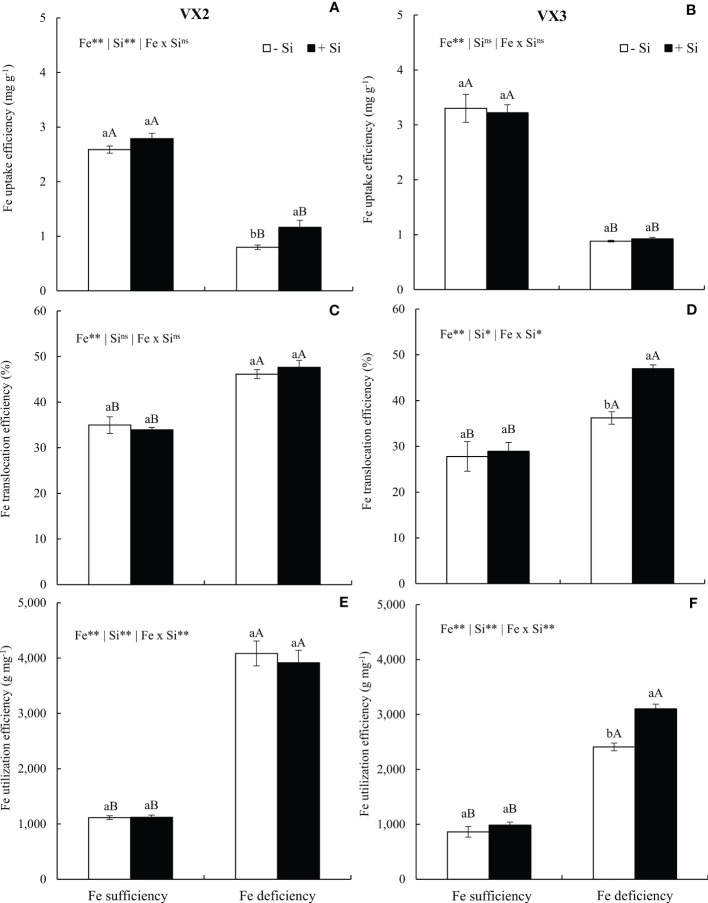
Uptake efficiency **(A, B)**, translocation **(C, D)**, and Fe utilization **(E, F)** in both sufficiency and deficiency of iron (Fe) conditions, associated with the presence (+Si) and absence (-Si) of silicon (Si) in plants of two cultivars of energy cane (VX2 and VX3). **, *significant at 1% and 5% probability levels, respectively; ^ns^not significant by the F-test. Lowercase letters indicate significant differences in relation to Si in the same Fe condition, while uppercase letters relates to Fe in the same condition of Si the Tukey’s test. Bars represent the standard error of means (n = 6).

The Fe uptake efficiency was reduced in the condition of Fe deficiency in both cultivars, but the provision of Si induced an increased efficiency in VX2 plants (**Figures 5A, B**). Efficiencies of translocations and utilization were both increased when the nutritional deficiency was imposed in the experiments, especially considering VX3 plants, which received Si (**Figures 5C–F**).

**Figure 6:**
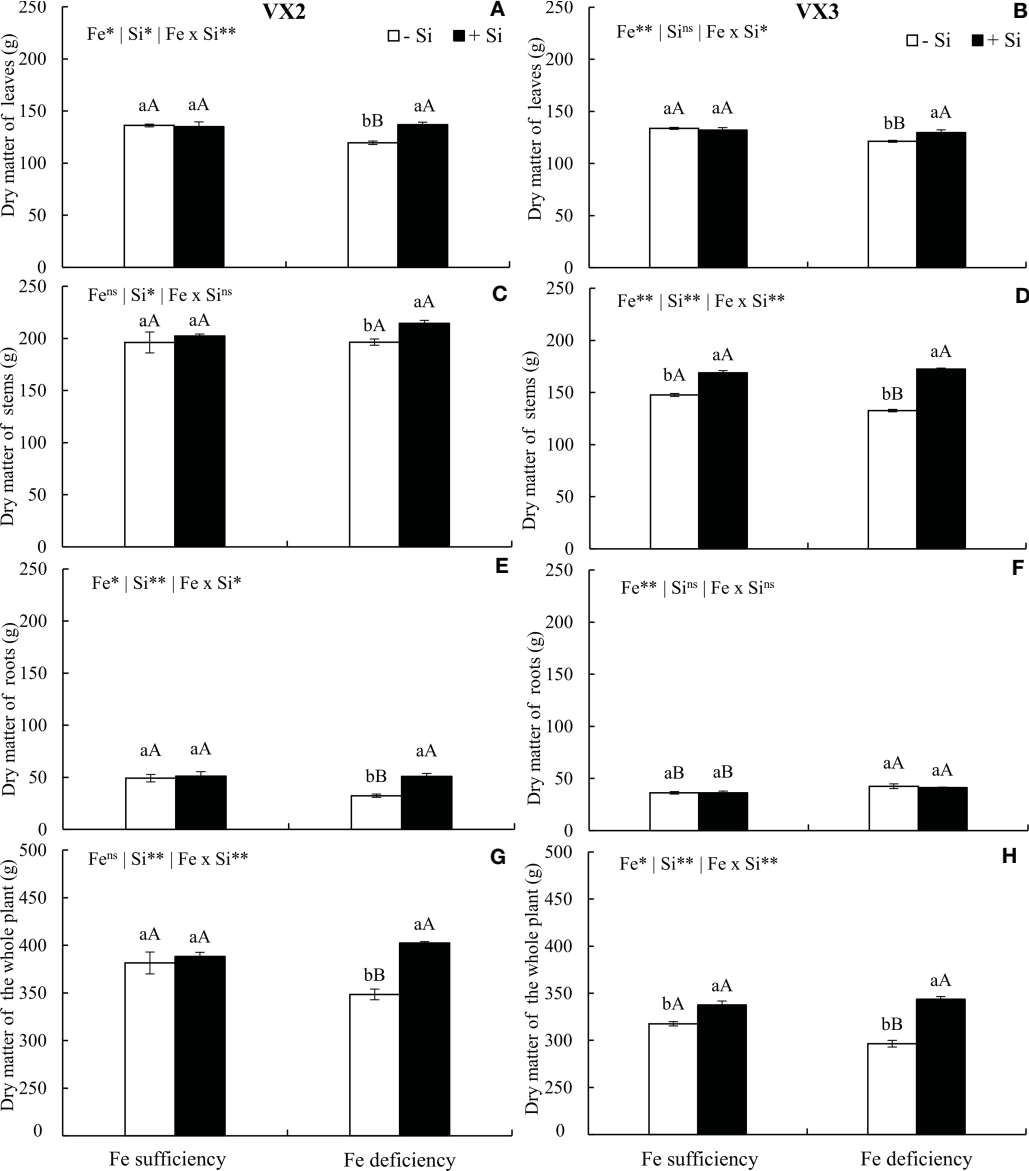
Dry matter of leaves **(A, B)**, stems **(C, D)**, roots **(E, F)** and whole plant **(G, H)** in both sufficiency and deficiency of iron (Fe) conditions, associated with the presence (+Si) and absence (-Si) of silicon (Si) in plants of two cultivars energy cane (VX2 and VX3). **, *significant at 1% and 5% probability levels, respectively; ^ns^not significant by the F-test. Lowercase letters indicate significant differences in relation to Si in the same Fe condition, while uppercase letters relates to Fe in the same condition of Si the Tukey’s test. Bars represent the standard error of means (n = 6).

The dry matter of leaves was reduced when plants were cultivated under Fe deficiency in both cultivars; however, supplying Si enabled the maintenance of leaves dry matter production, which was similar to the results observed in plants cultivated under sufficiency conditions (**Figures 6A, B**). Fe deficiency caused a reduced dry matter production of stems only in VX3 plants, but the presence of Si increased the stems dry matter in the condition of Fe deficiency in both cultivars (**Figures 6C, D**). The dry matter production of roots was reduced under Fe deficiency only in VX2 plants, and in this condition, Si contributed for the maintenance of the mass in a similar threshold to that observed in plants under sufficiency of the micronutrient (**Figures 6E, F**).

The dry matter of the whole plant was also affected by the nutritional deficiency of Fe, being reduced by 10% and 7% in VX2 and VX3, respectively, in comparison to the condition of sufficiency. Nevertheless, the supply of Si incremented its dry matter in 16% in comparison to the condition of absence of Si, in both cultivars (**Figures 6G, H**).

**Figure 7:**
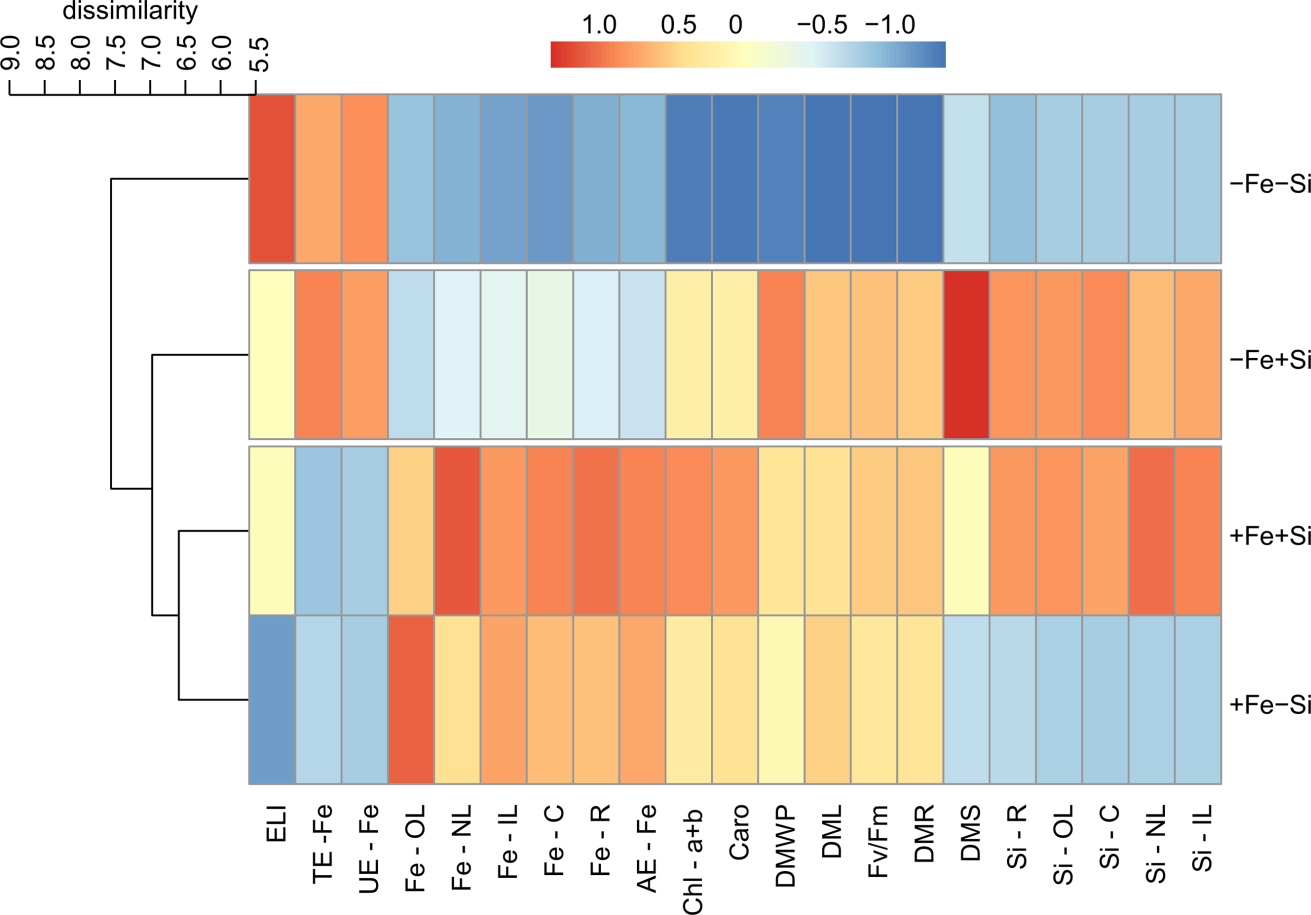
Hierarchical cluster analysis with standardized data of silicon (Si) and iron (Fe) accumulations in new (NL), intermediate (IL) and old leaves (OL), in stems (C) and roots (R); electrolyte leakage index (ELI), total chlorophyll content (Chl a+b), carotenoids (Caro), quantum efficiency of FSII (Fv/Fm); uptake efficiency (AE), translocation (TE), and utilization (UE) of Fe; dry matter of leaves (DML), stems (DMS), roots (DMR) and whole plant (DMWP) in both sufficiency and deficiency of Fe conditions, associated with the presence (+Si) and absence (-Si) of Si in plants of cultivar energy cane (VX2).

**Figure 8:**
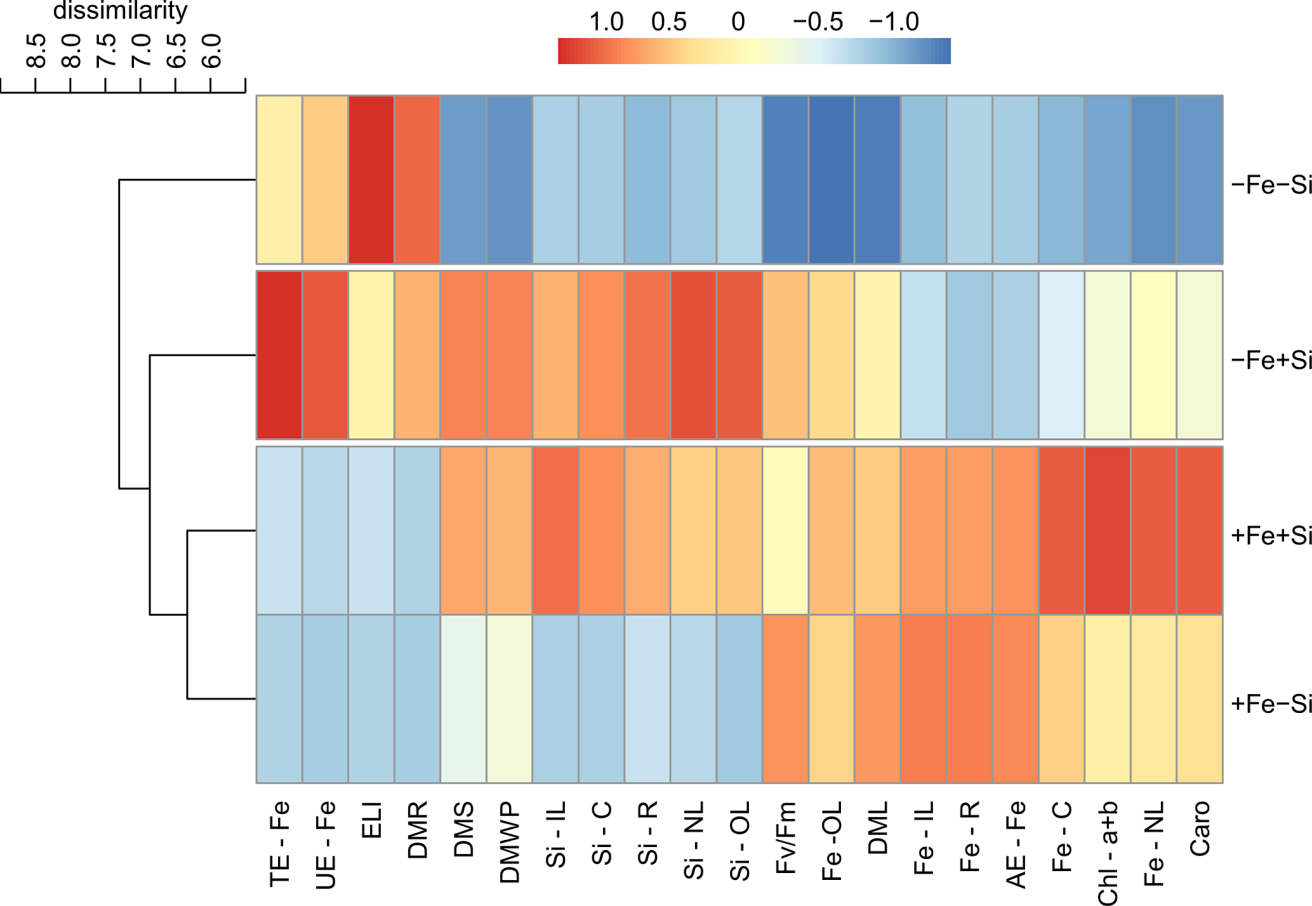
Hierarchical cluster analysis with standardized data of silicon (Si) and iron (Fe) accumulations in new (NL), intermediate (IL) and old leaves (OL), in stems (C) and roots (R); electrolyte leakage index (ELI), total chlorophyll content (Chl a+b), carotenoids (Caro), quantum efficiency of FSII (Fv/Fm); uptake efficiency (AE), translocation (TE), and utilization (UE) of Fe; dry matter of leaves (DML), stems (DMS), roots (DMR) and whole plant (DMWP) in both sufficiency and deficiency of Fe conditions, associated with the presence (+Si) and absence (-Si) of Si in plants of cultivar energy cane (VX3).

The cluster analysis revealed that the effects caused by the presence of Si resulted in the classification of plants into two groups. For both cultivars, plants grown under Fe deficiency and +Si were grouped with plants under Fe sufficiency in both conditions of Si supply but separated from Fe deficiency in -Si. After standardizing the data, it was evident that the effects of Si supply in plants of cultivar VX2 started with an increased accumulation of Si and Fe, causing a reduction in the physiological damage related to oxidative stress, as seen in the lower index of electrolyte leakage index, as well as in the photosynthetic capacity, involving the highest content of chlorophyll and carotenoids, in addition to the quantum efficiency of PSII. However, in this cultivar, the nutritional efficiencies of Fe did not show a very evident effect (**Figure 7**). Regarding the cultivar VX3, the beneficial effects of Si in plants deficient in Fe were seen both in the physiological variables and in the increased nutritional efficiencies, especially in the translocation and use of Fe. However, Si provided an increased dry matter production in Fe-deficient plants of both cultivars (**Figure 8**).

## 4 Discussion

Plants of the Poaceae family including energy cane usually have a high demand for Fe (Carrasco-Gil et al., 2018). The results obtained in this study evidenced for the first time the damage of Fe deficiency in plants of energy cane. This shows that this is a sensitive species to such a nutritional imbalance and what the differences in aptitudes do not interfere in the Fe tolerance mechanisms, and both cultivars evaluated can be damaged when cultivated in an environment with Fe deficiency.

The harms triggered by Fe deficiency occurred because of a reduced buildup of this nutrient in the plants, implying in the accumulation of reactive oxygen species (ROS) in leaves, as an increased cellular electrolyte leakage index was observed in both cultivars of energy cane. This increased oxidative stress promoted by Fe deficiency occurred because the photosystem II (PS-II) contains Fe proteins and loses its efficiency in Fe-deprived plants due to the low supply of photosynthetic electrons (Bertamini et al., 2002), thus decreasing the energy to optimize the photosynthetic process.

Additionally, there was a decrease in the efficiency of the enzymatic or non-enzymatic defense system of plants, which can be emphasized by the reduction of carotenoids (non-enzymatic antioxidant compounds) in the condition of Fe deficiency in the absence of Si. The defense enzymatic system may also have been affected considering a possible decrease in activity antioxidant enzymes, such as ascorbate peroxidase and catalase, which are dependent on Fe because these contain the *heme* group in its structure (Kallala et al., 2021). Moreover, Fe is required as a catalyst for electron transfer reactions by many antioxidant enzymes, such as superoxide dismutase (SOD). Thus, such enzymes use Fe to catalyze H_2_O_2_ (hydrogen peroxide) and OH^●^ (hydroxyl radical), which are either reduced to H_2_O (antioxidant activity) or oxidized as O_2_^●-^ (superoxide anion radical), with the concomitant reduction from Fe(III) to Fe(II) (Tewari et al., 2013). These combined effects caused damage to the metabolism of plants under iron deficiency and without Si, inducing oxidative stress scenarios.

While favoring the cellular oxidative stress, the Fe deficiency in the absence of Si reduced the chlorophyll contents of plants, due to its degradation of foliar tissues, and compromised the biosynthesis of this pigment (Becker et al., 2020; de Mello Prado, 2021). This effect of Fe deficiency limited the photosynthetic efficiency, as it reduced the Fv/Fm ratio, which indicates an impairment of the photosystem II (Bertamini et al., 2002; Yadavalli et al., 2012), and consequently the production of dry matter in both cultivars.

In view of the results obtained in this study, it is evident that the use of strategies for the application of Si aiming to mitigate damages caused by Fe deficiency in energy cane becomes necessary. The benefit of Si application in Fe deficiency condition began with the increment of this element in the plants, seen that energy cane plants belong to the Poaceae family, which is considered an accumulator of Si by having specific chargers for the root uptake of this element, showing Si contents greater than 10 g kg^-1^ (Ma and Takahashi, 2002). This fact was evidenced in this research because the supply of Si induced an average Si content of 10.83 and 10.48 g kg^-1^ in cultivar VX2 and 11.91 and 12.35 g kg^-1^ in cultivar VX3 for the Fe sufficiency and deficiency conditions, respectively. The optimal uptake of Si by plants was also due to the applied solution at an adequate concentration (2.5 mmol L^-1^), which avoided the polymerization that occurs when Si is found at concentrations above 3.0 mmol L^-1^ (Birchall, 1995), in addition to the acid pH of the nutrient solution used in the experiment that resulted in the production of monomeric species of Si (H_4_SiO_4_) (Kudryavtsev and Figovsky, 2016), favoring its uptake.

It is noteworthy that the plants were initially fertilized with Si *via* fertirrigation applied prior to transplanting during the seedling formation phase. Thus, it can be inferred that the previous supply of Si to the plants has contributed to alleviating the damages caused by Fe deficiency, which occurs predominantly in alkaline soils (Abbaszadeh-Dahaji et al., 2020). Associated with this, the supply of Si after transplantation in a pH-adjusted nutrient solution has added the effects by inducing immediate Si uptake. However, in an alkaline solution, Si species predominates in the form of monovalent (H_3_SiO_4_^-^) and divalent (H_2_SiO_4_^-2^) anions. These monomeric species can be absorbed, mitigating the deficiency of Fe, in case the concentration is maintained up to 3 mmol L^-1^ of Si, considering that all monomeric species, regardless of pH value, induce the formation of polysilicon acid forming silanol groups (Si-O-Si) with polymerization, and are no longer absorbed by plants (Kudryavtsev and Figovsky, 2016).

The increased uptake of Si in plants of both cultivars cultivated under Fe deficiency promoted an accumulation of Fe in all parts of the plants, except in old leaves in cultivar VX2 and roots in cultivar VX3. A modification in the order of accumulation of this micronutrient in the plants under Fe deficiency and +Si was observed, with higher amounts being found in intermediate leaves (7.48 mg per plant), in comparison to old leaves (6.69 mg per plant) in VX2. This result was different to the ones observed in Fe deficiency and -Si plants, which displayed a higher accumulation of Fe in old leaves (5.93 mg per plant) in comparison to intermediate leaves (3.27 mg per plant). This may have occurred because of Si decreases callus formation in the conducting vessels, thus facilitating the movement of Fe in the phloem (Doncheva et al., 2009). In addition, may have occurred the increase in the biosynthesis of Fe-mobilizing compounds such as citrate in roots and leaves and catechins in roots favored by Si seems to be of great importance for improving the redistribution and utilization of Fe (Chaiwong et al., 2020).

The higher Fe flow in the phloem enabled an increased accumulation of this micronutrient in intermediate leaves, which are more active in terms of photosynthesis, in comparison to old ones. This indicates that Si has a role in maintaining Fe homeostasis in plants through the formation of Fe-Si complexes and through the maintenance of the redox potential in the solution of the root apoplast and the xylem sap, which favors Fe transport through the xylem and its subsequent accumulation in the shoot tissues (Lata-Tenesaca et al., 2023). The beneficial effect of Si in the accumulation of Fe was evidenced for the first time in energy cane plants, which was also reported in barley (Nikolic et al., 2019) and sorghum (Teixeira et al., 2020a).

With an increased Fe uptake mediated by Si, the stress associated to nutritional deficiency was reduced in both cultivars of energy cane. From this result, it can be inferred that the greater accumulation of Fe in the plants has induced the activation of the antioxidant system, as this element composes antioxidant enzymes (Gratão et al., 2005). In addition, the contribution for the increased concentration of carotenoid antioxidant compounds was relevant (Havaux, 2014) acts by reducing the formation of ROS. These effects of Si in plants with micronutrient deficiencies favored the membrane’s integrity, as observed by the reduce cellular electrolyte leakage index, which was also verified in sorghum plants submitted to Fe (Teixeira et al., 2020a) and Mn (De Oliveira et al., 2019) deficiencies. The effect of Si in inducing the activation of the antioxidant system to maintain cell’s integrity and homeostasis in Fe-deficient plants was reported for cucumber (Bityutskii et al., 2014), soybean (Gonzalo et al., 2013), rice (Martín-Esquinas and Hernández-Apaolaza, 2021) and barley (Nikolic et al., 2019).

The antioxidant benefit of Si in plants cultivated under Fe deficiency reflected in the preservation of photosynthetic pigments (chlorophyll and carotenoids), and when combined with the higher concentration of Fe in the plants, induced chlorophyll synthesis, as this micronutrient composes the precursor of this pigment (de Mello Prado, 2021).

Energy cane plants grown under Fe deficiency that were cultivated with the nutrient solution containing Si increased the Fe uptake efficiency, especially in the cultivar with the highest capacity of producing fibers (VX2), which reflected in a 110% increase in the accumulation of this micronutrient in comparison to VX3. However, in the cultivar with the lowest fiber production and higher sucrose production (VX3), the effect of Si stood out in producing an increased efficiency of translocation and utilization of Fe.

Is became evident that in both cultivars of energy cane under Fe deficiency, the application of Si increased the photosynthesis efficiency (Fv/Fm) and favored dry matter production of plants. Thus, the hypothesis related to the mechanisms of Si in mitigating Fe deficiency can accepted, considering the accumulation of Fe in plants and the nutritional efficiency, especially in relation to the uptake observed in VX2 and the translocation and utilization in VX3. These results were related to the reduction of the cellular electrolyte leakage index, as well as to the increased concentration of carotenoids, which consequently increased the chlorophyll content, photosynthetic efficiency and dry matter production. Furthermore, it was found that the differences in aptitudes did not interfere in the mechanisms of Fe tolerance, and both cultivars were damaged when grown in an environment with moderate Fe deficiency. When Si was added, the responses of both cultivars were efficient, with a single difference being observed in the mechanisms of action of the Si, dismissing the second hypothesis that cultivar aptitude affects Fe deficiency tolerance.

This study proposes a new strategy based on the use of Si applied via fertirrigation for the cultivation of energy cane, in a condition of Fe deficiency, which occurs in several regions of the world, to increase the production of renewable energy with improved sustainability. Silicon can be used as a modulating agent of physiological and nutritional defense mechanisms that contribute to the mitigation of Fe deficiency in two energy cane cultivars.

It is now important to expand these studies to other species with mechanisms contrasting in nature for absorption of Fe in more than one growing season, to increase knowledge about the interaction of Si and Fe in plant physiology and nutrition.

## 5 Conclusion

Both cultivars of energy cane used in this study were sensitive to Fe deficiency, demonstrating that aptitudes do not interfere in the tolerance to Fe deficiency. In the absence of Si, Fe deficiency hampered plants growth by causing stress, by degrading its pigments and jeopardizing the photosynthetic efficiency. The supply of Si mitigated Fe deficiency in these cultivars by increasing Fe accumulation and reducing plants’ stress condition, thus favoring both nutritional and photosynthetic efficiencies, especially in relation to the uptake in VX2 cultivar and the translocation and utilization in VX3 cultivar, as well as dry matter production.

Si by modulating physiological and nutritional mechanisms, mitigates Fe deficiency in two energy cane cultivars.

## Data availability statement

The raw data supporting the conclusions of this article will be made available by the authors, without undue reservation.

## Author contributions

GT did the conceptualization, carried out the data curation and formal analysis, investigated the data, performed the methodology, wrote the original draft of the manuscript, and wrote, reviewed, and edited the manuscript. RP did the conceptualization, carried out the funding acquisition, project administration, resources, performed the methodology, supervised the data, and wrote, reviewed, and edited the manuscript. AR carried out the formal analysis, investigated the data, performed the methodology, and wrote the original draft of the manuscript. MP investigated the data, performed the methodology. CA investigated the data, performed the methodology. All authors contributed to the article and approved the submitted version.
